# Nanophotonics in support of Ukrainian Scientists

**DOI:** 10.1515/nanoph-2023-0360

**Published:** 2023-07-10

**Authors:** Nader Engheta, Nikolay I. Zheludev, Dennis Couwenberg

**Affiliations:** University of Pennsylvania, PA, Pennsylvania, USA; Optoelectronics Research Centre & Centre for Photonic Metamaterials, University of Southampton, Southampton, UK; Marie Heinekenplein 404, 1072 ML, Amsterdam, The Netherlands

On February 24, 2022, the world was shocked by the Russian invasion of Ukraine. Since then, many lives have been lost, people have been injured, and families have become refugees. The National Academies of the G7 states, and many other scientific and academic organizations all over the world, issued statements condemning the aggression on Ukraine.

In solidarity with the people of Ukraine the Publishing Editor and De Gruyter, the Publisher of “Nanophotonics”, have initiated a project titled “Nanophotonics in support of Ukrainian Scientists”.

Prominent scientists have contributed thirty-three papers to the special issue, covering a diverse range of topics related to the burgeoning discipline of nanophotonics. The Publisher is donating all of the open access article fees from this special issue to support Ukrainian scientists. Together with a donation from one of the authors of the special issue, Michael Berry, Foreign Member of the Academy of Sciences of Ukraine, this amounts to Euro 80,575.00. The money will be donated to support scientists who are located in the Ukraine and have had to relocate.

We believe that the devastating invasion will end, that peace and prosperity will return to Ukraine in its historic borders and Ukraine will rejoin the international research community. The special issue is demonstrating the power of science to bring all of us together in a difficult moment in history.

**Nader Engheta**

**Nikolay I. Zheludev** Guest Editors

**Dennis Couwenberg** Publishing Editor

